# Humanized mouse model: Hematopoietic stemcell transplantation and tracking using short tandem repeat technology

**DOI:** 10.1002/iid3.317

**Published:** 2020-06-11

**Authors:** Lia Walcher, Nadja Hilger, Anja K. Wege, Franziska Lange, U. Sandy Tretbar, André‐René Blaudszun, Stephan Fricke

**Affiliations:** ^1^ Department of Immunology Fraunhofer Institute for Cell Therapy and Immunology Leipzig Germany; ^2^ Department of Gynecology and Obstetrics University Medical Center Regensburg Regensburg Germany; ^3^ Department of Diagnostics Fraunhofer Institute for Cell Therapy and Immunology Leipzig Germany

**Keywords:** adult mobilized stem cells, cord blood‐derived stem cells, hematopoietic stem cells, humanized mouse model, short tandem repeat

## Abstract

**Introduction:**

Models of mice carrying a human immune system, so‐called humanized mice, are used increasingly as preclinical models to bridge the gap between model organisms and human beings. Challenges of the humanized mouse model include finding suitable sources for human hematopoietic stem cells (HSC) and reaching sufficient engraftment of these cells in immunocompromised mice.

**Methods:**

In this study, we compared the use of CD34^+^ HSC from cord blood (CB) vs HSC from adult mobilized peripheral blood. Furthermore, we developed a simple and highly specific test for donor identification in humanized mice by applying the detection method of short tandem repeats (STR).

**Results:**

It was found that, in vitro, CB‐derived and adult HSC show comparable purity, viability, and differentiation potential in colony‐forming unit assays. However, in vivo, CB‐derived HSC engrafted to a significantly higher extent in NOD.Cg‐Prkdc^scid^IL2rγ^tm1Wjl^/SzJ (NSG) mice than adult HSC. Increasing the cell dose of adult HSC or using fresh cells without cryopreservation did not improve the engraftment rate. Interestingly, when using adult HSC, the percentage of human cells in the bone marrow was significantly higher than that in the peripheral blood. Using the STR‐based test, we were able to identify and distinguish human cells from different donors in humanized mice and in a humanized allogeneic transplantation model.

**Conclusion:**

From these findings, we conclude that adult mobilized HSC are less suitable for generating a humanized immune system in mice than CB‐derived cells.

## Graphical abstract

In this study, the authors present new insights into the humanized immune system mouse model. CB‐derived hematopoietic stem cells (HSC) are shown to engraft in NSG mice to a significantly higher extent than adult HSC. Furthermore, a simple and highly specific test method for the tracking of the human donor(s) in humanized mice is presented.

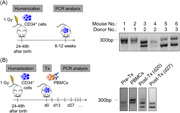

**Abbreviations**
7‐AAD: 7‐aminoactinomycin D
CB: cord blood
CFU: colony‐forming unit
FSC: forward scatter
G‐CSF: granulocyte colony‐stimulating factor
HSC: hematopoietic stem cell
CD: cluster of differentiation
hu: human
mu: murine
NOG: NOD/Shi‐scid/IL‐2Rγ^null^
NSG: NOD.Cg‐Prkdc^scid^IL2rγ^tm1Wjl^/SzJ
PB: peripheral blood
PBMC: peripheral blood mononuclear cell
PBS: phosphate buffered saline
PCR: polymerase chain reaction
SD: standard deviation
SSC: sideward scatter
STR: short tandem repeat
Tx: transplantation

## 1 INTRODUCTION

Mouse models are a widely used option for basic research and preclinical assessment.^1^ The model of humanized mice, that is, mice carrying a human immune system, enables analyses of the human immune system and its complex interactions.^2^, ^3^ There are three main procedures to generate humanized mice: transplantation (Tx) of peripheral blood mononuclear cells (PBMC), transfer of fetal liver and thymus fragments together with bone marrow hematopoietic stem cells (HSC), and injection of cluster of differentiation(CD)34^+^ HSC.^2^, ^3^ As the PBMC‐model is prone to graft‐vs‐host disease development^4^ and the bone‐marrow‐liver‐thymus‐model is limited by the availability of fetal tissue and ethical concerns, the HSC‐humanized model has become the most frequently used model.^2^

A commonly used method for humanization was published by Pearson et al,^5^ wherein neonatal immunodeficient mice are sublethally irradiated (1 Gy) and injected intrahepatically with CD34^+^ cells isolated from human umbilical cord blood (CB). Previously, bone marrow and mobilized peripheral blood (PB) of adults have been used as alternative HSC sources, however, a significant reduction in humanization efficiency was observed compared to the humanization efficiency of CB‐derived HSC.^6^, ^9^ Nevertheless, a humanized mouse model based on adult cells is desirable, especially under the aspect of personalized medicine, as it could be a platform for individualized, patient‐specific research. Therefore, we re‐evaluated the usage of adult mobilized HSC with a specific focus on two relevant parameters, namely, cryopreservation and cell dose, and analyzed the in vivo engraftment in comparison to CB‐derived HSC.

Another important aspect of humanized mouse models is the identification of the human donor. Human cells can be detected by measuring the human CD45 (huCD45) expression via flow cytometry. However, in certain experimental settings (eg, humanized allogeneic Tx models), human immune cells from two different donors are present, and therefore, more specific methods are required.^10^, ^11^ Here, we show a polymerase chain reaction (PCR)‐based assay that enables a highly specific identification of human donors via a short tandem repeat (STR) motif, the SE33 locus.^12^ As the STR pattern is unique for every individual, it is possible to distinguish mice that were humanized from different donors. Furthermore, the assay allows the simultaneous detection of cells from two different human donors (chimerism) in a humanized Tx model.

## 2 MATERIALS AND METHODS

### 2.1 Humanization of NSG mice

NOD.Cg‐PrkdcscidIL2rγtm1Wjl/SzJ (NSG) mice were obtained from The Jackson Laboratory and were bred and kept under special pathogen‐free conditions. Neonatal NSG mice (24‐48 hours after birth, male and female) were irradiated with 1 Gy X‐Rayirradiation (SARRP; Xstrahl, Germany). After a minimum of 4 hours of recovery time, 2 × 10^5^ or 4 × 10^5^ CD34^+^ HSC were injected intrahepatically. The transplantation of PBMC was carried out by intravenous injection of 2 × 10^7^ cells (preincubated with or without anti‐human CD4 antibody MAX.16H5 IgG_4_ to suppress the graft‐vs‐host reaction potentially mediated by the graft, a concept which we already described earlier^4^, ^13^, ^14^). PB samples were obtained by retrobulbar bleeding under anesthesia. Bone marrow samples were obtained by femoral aspiration *post mortem*. All animal experiments were performed according to the national guidelines for animal experiments and were approved by the local animal protection committee (Landesdirektion Sachsen).

### 2.2 Primary cells and ethical statement

CB was obtained from healthy full‐term newborns. Adult HSC were isolated from leukapheresis products of granulocyte colony‐stimulating factor (G‐CSF) mobilized, healthy donors (Cellex, Germany). PBMC isolated from buffy coats were purchased from a blood bank (Institute for Transfusion Medicine, University of Leipzig, Germany). All donors gave informed consent and all experiments were approved by the local ethics committee.

### 2.3 Isolation of CD34^+^ HSC

Mononuclear cells were isolated from blood products by Ficoll‐Paque density gradient centrifugation (Biocoll; Biochrom, Germany). CD34^+^ HSC were isolated by positive magnetic selection (human CD34 MicroBead Kit UltraPure; Miltenyi Biotec, Germany) according to the manufacturer's instructions. Cryopreservation was performed in human serum supplemented with 10% dimethyl sulfoxide (both from Sigma‐Aldrich, Germany) at a cooling rate of 1°C/min.

### 2.4 Flow cytometry

For flow cytometric analysis, the following antibodies were used: PE‐labeled anti‐huCD34 (clone 563), V500‐labeled anti‐huCD45 (clone HI30), PE‐labeled anti‐murine (mu)CD45 (clone 30‐F11), 7‐aminoactinomycin D (7‐AAD; all from BD Biosciences, Germany). Antibody staining was performed protected from light for 20 minutes at room temperature following one washing step with phosphate buffered saline (PBS). 7‐AAD was added to the samples directly before measurement. Acquisition was performed using BD FACSCantoII and data was analyzed using BD FACSDiva software. Recorded events were gated for single cells (FSC‐H × FSC‐A), then for living cells (FSC‐A × SSC‐A), and finally for the respective marker, except for the 7‐AADgate, which was applied directly to the single‐cell gate.

### 2.5 Colony‐forming unit (CFU)‐Assay

For CFU analysis, a MethoCult CFU‐assay kit was used (Stemcell Technologies, France) according to manufacturer's instructions.

### 2.6 PCR

DNA was extracted from 20 to 30 µL of PB using the E.Z.N.A. Blood DNA Mini Kit (Omega Bio‐tec). STR motifs were amplified by PCR and evaluated by gel electrophoresis using 3% agarose gels. A primer pair for SE33 (forward sequence: AATCTGGGCGACAAGAGTGA; reverse sequence: ACATCTCCCCTACCGCTATA; purchased from MWG Eurofins) and the following amplification protocol was used: 35 cycles of denaturation (30 seconds at 95°C), annealing (30 seconds at 50°C) and elongation (60 seconds at 72°C).

### 2.7 Statistical analysis

Statistical tests were performed using GraphPad Prism 6 software (GraphPad Software, Inc.). All data were analyzed for normal distribution using D'Agostino and Pearson omnibus normality test. In the case of non‐normally distributed data, the Mann‐Whitney test was used. All values are indicated as mean ± standard deviation (SD) and statistical significance is symbolized by asterisks (*P < .05, **P < .01, ***P < .001, and ****P < .0001).

## 3 RESULTS AND DISCUSSION

### 3.1 Adult and CB‐derived HSC show similar stability and differentiation potential in vitro

**Figure 1:**
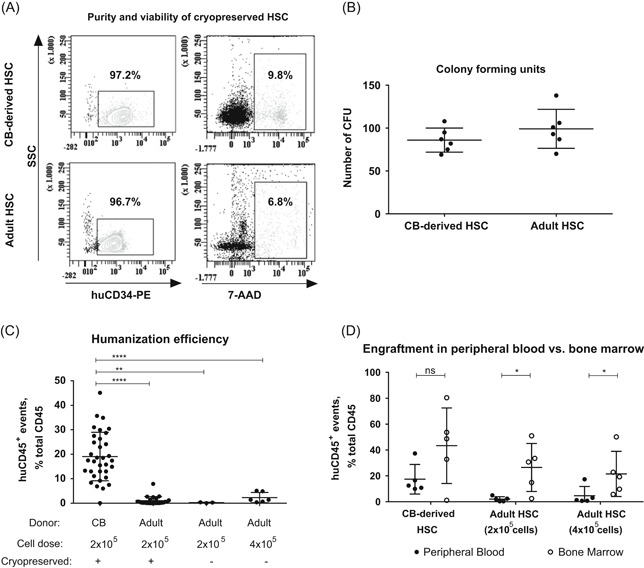
Qualitative and functional assessment of CB‐derived and adult HSC in vitro and humanization efficiency in NSG mice in vivo. A, Flow cytometric analysis of CB‐derived and adult HSC after CD34‐magnetic separation and cryopreservation. Representative flow cytometry plots are shown, in total between n = 3 and n = 18 biological replicates were analyzed. B, Colony formation of CB‐derived and adult HSC determined by CFU‐assay in methylcellulose (n = 3 biological replicates, each in n = 2 technical replicates). C, Flow cytometric analysis of humanization efficiency 8–12 weeks after humanization with CB‐derived HSC (n = 34 mice humanized from n = 18 donors in n = 6 independent experiments); adult, cryopreserved HSC (n = 24 mice humanized from n = 8 donors in n = 5 independent experiments) or adult, fresh HSC at doses of 2 × 10^5^ (n = 3 mice humanized from n = 2 donors in n = 1 independent experiment) or 4 × 10^5^ cells (n = 6 mice humanized from n = 3 donors in n = 2 independent experiments). D, Flow cytometric analysis of huCD45^+^ events in peripheral blood and bone marrow are shown of mice humanized with CB‐derived HSC (n = 5 mice humanized from n = 3 donors in n = 2 independent experiments) or adult HSC at doses of 2 × 10^5^ (n = 5 mice humanized from n = 4 donors in n = 2 independent experiments) or 4 × 10^5^ cells (n = 5 mice humanized from n = 2 donors in n = 2 independent experiments). In the flow cytometric experiments, recorded events were gated for single cells (FSC‐H × FSC‐A), then for living cells (FSC‐A × SSC‐A), and lastly, for the respective marker, except for the 7‐AADgate, which was applied directly to the single‐cell gate. The following antibodies were used: PE‐labeled anti‐huCD34, V500‐labeled anti‐huCD45, PE‐labeled anti‐muCD45, 7‐AAD. Values are indicated as mean ± SD, asterisks indicate the statistical significance of the Mann‐Whitney test. 7‐AAD, 7‐aminoactinomycin D; CB, cord blood; CFU, colony‐forming unit; HSC, hematopoietic stem cell; FSC, forward scatter; NSG, NOD.Cg‐Prkdc^scid^IL2rγ^tm1Wjl^/SzJ; SSC, sideward scatter. *P < .05, **P < .01, and ****P < .0001

CD34^+^ adult and CB‐derived HSC were isolated by magnetic‐activated cell sorting. The purity and viability of the cells were analyzed by flow cytometry. Both cell types showed comparable purity (97.24 ± 2.30% and 96.82 ± 3.50% CD34^+^ events of life gate for CB‐derived and adult HSC, respectively; *P* = .7748) and viability (percentages of dead cells: 11.17 ± 4.90% and 6.83 ± 6.10% 7‐AAD^+^ events of single cells for CB‐derived and adult HSC, respectively; *P* = .2619). Representative plots are displayed in Figure 1A. After isolation, the CD34^+^ HSC were assessed in CFU assays. HSC from adult donors showed the same colony‐forming potential as CB‐derived HSC (86.00 ± 14.09 CFU vs 99.17 ± 22.78 CFU, respectively; see Figure 1B).

### 3.2 Adult HSC cause a lower humanization efficiency than CB‐derived HSC

Neonatal NSG‐mice were humanized with CD34^+^ human HSC isolated either from cryopreserved CB (n = 34), from cryopreserved (n = 24), or fresh adult leukapheresis products (n = 3 for 2 × 10^5^ applied cells, n = 6 for 4 × 10^5^ applied cells). Eight to twelve weeks after humanization, the humanization efficiency was determined by flow cytometric analysis of huCD45^+^ events in the PB (Figure 1C). Immune reconstitution of mature cells was verified by flow cytometry and revealed mainly B‐cell reconstitution (73.26 ± 8.79% CD3^‐^CD19^+^ cells), followed by T‐cells (7.43 ± 3.67% CD3^+^CD4^+^ cells and 11.97 ± 4.77% CD3^+^CD8^+^ cells), NK‐cells (1.37 ± 0.40% CD56^+^ cells) and monocytes/macrophages (0.32 ± 0.18% CD14^+^ cells) (n = 10 mice humanized with CB‐derived HSC, data not shown). This trend is congruent with data previously reported by others.^15^, ^16^ Using CB‐derived HSC resulted in a significantly higher degree of humanization (19.06 ± 9.91%) compared to cryopreserved adult HSC (1.03 ± 1.71%; *P* < .0001; Mann‐Whitney Test). We aimed to optimize the protocol by avoiding quality loss due to cryopreservation and hence used fresh leukapheresis product. However, the humanization efficiency was still significantly lower compared to the humanization efficiency of CB‐derived HSC (0.27 ± 0.15%; *P* = .0012; Mann‐Whitney Test). Also, an increase in the applied cell number did not improve the humanization efficiency (2.32 ± 2.12%; *P* < .0001 compared to CB‐derived HSC; Mann‐Whitney Test).

Concluding, only the standard protocol using CB‐derived HSC was suitable for an efficient humanization. Although in vitro stemcell properties were comparable between CB‐derived and adult HSC, there were significant differences in the in vivo engraftment. This suggests that neonatal and adult HSC have different properties considering the engraftment and differentiation potential in mice. A possible explanation for this could be the different proportion of CD34^+^CD38^−^ cells observed between CB‐derived and adult HSC.^17^, ^18^ However, clinically, mobilized adult cells are widely used for autologous and allogeneic HSC Tx and have even been shown to engraft significantly earlier than CB‐derived HSC.^19^ The differences observed in the humanized mouse model is, therefore, likely caused by the absence of certain human factors in mice. Interestingly, immunodeficient mouse strains modified by knock‐in of human cytokines showed higher engraftment of adult HSC compared to NSG mice, but still, the levels of huCD45^+^ events in the PB did not exceed the 20% threshold.^8^ A study by van der Loo et al^20^ demonstrated that even at very high doses of mobilized adult HSC (up to 5 × 10^7^ CD34^+^ cells) only a minor share of NOD/SCID mice show more than 20% human cells in the PB. On this basis, the authors calculated the frequency of NOD/SCID‐repopulating cells as 1 in 1.7 × 10^6^ CD34^+^ cells of adult mobilized HSC.^20^

Previously, two different studies have directly compared the generation of humanized mice using adult mobilized HSC vs using CB‐derived HSC.^6^, ^7^ Matsumura et al^6^ showed that the intravenous injection of HSC into 9‐week‐old NOG (NOD/Shi‐scid/IL‐2Rγ^null^)‐mice resulted in engraftment levels of 10.7 ± 5.8% and 47.5 ± 39.7% huCD45 in the PB when using adult mobilized cells or CB‐derived cells, respectively. Similar results were obtained by Lepus et al,^7^ who employed the intrahepatic injection protocol of neonatal NSG mice and detected an average of 12.7% (adult HSC) or 51.8% (CB‐derived HSC) huCD45 in the PB. It is important to mention, that in both studies a much higher cell dose of adult HSC was applied compared to the dose of CB‐derived cells.^6^, ^7^ In our study, constant parameters were used in both protocols, to enable a direct comparison. Furthermore, to our knowledge, this is the first study that compares fresh and cryopreserved adult HSC regarding their humanization potential. Surprisingly, the usage of fresh adult cells resulted in no improvement in the humanization degree. In an earlier study by Scholbach et al,^15^ the engraftment levels of fresh vs cryopreserved CB‐derived HSC into NSG mice were compared. Slightly higher levels of huCD45 were reached after humanizing mice with fresh cells (20.8%) than after humanizing mice with cryopreserved cells (11%),^15^ indicating a mild benefit for using fresh CB‐derived cells, that was not observable for adult HSC in our study.

### 3.3 Adult HSC engraft in the bone marrow but human immune cells are not detectable in the PB

Mice that did not reach a sufficient humanization degree (< 20% huCD45^+^ events of total CD45^+^ events) were killed 13 weeks after humanization. Cells from the PB and the bone marrow were analyzed by flow cytometry for expression of huCD45 and muCD45. Interestingly, acceptable human engraftment levels could be found in the bone marrow of mice humanized with mobilized HSC and overall, human engraftment in the bone marrow was higher compared to the levels detected in the PB of all humanized mice (Figure 1D). In bone marrow samples, the levels of huCD45 were 43.30 ± 29.16% in CB‐derived HSC transplanted mice (n = 5), 26.50 ± 18.52% in adult HSC transplanted mice using 2 × 10^5^ cells (n = 5) and 21.52 ± 17.42% in adult HSC transplanted mice using 4 × 10^5^ cells (n = 5). In the PB samples, however, levels of huCD45 were 17.42 ± 11.39% for mice humanized with CB‐derived HSC (n = 5), 2.04 ± 1.89% for 2 × 10^5^ adult HSC (n = 5) and 4.64 ± 7.19% for 4 × 10^5^ adult HSC (n = 5). A statistically significant difference was observed for adult HSC at both doses (*P* = .0159 for 2 × 10^5^ cells and *P* = .0317 for 4 × 10^5^ cells, Mann‐Whitney test). This suggests, that while the HSC of adult donors engraft well in the bone marrow, differentiated human immune cells are not manifested in the periphery. A possibility to stimulate egress of cells from the bone marrow is treating animals with human G‐CSF or G‐CSF in combination with stem cell factor, which was previously shown to result in a slight increase of human cells in the PB.^20^ Analysis of lymphoid precursor cells in the thymus and bone marrow could give insights about possible developmental blocks in hematopoiesis and should be included in future studies.

### 3.4 PCR analysis of STR enables specific donor identification

**Figure 2:**
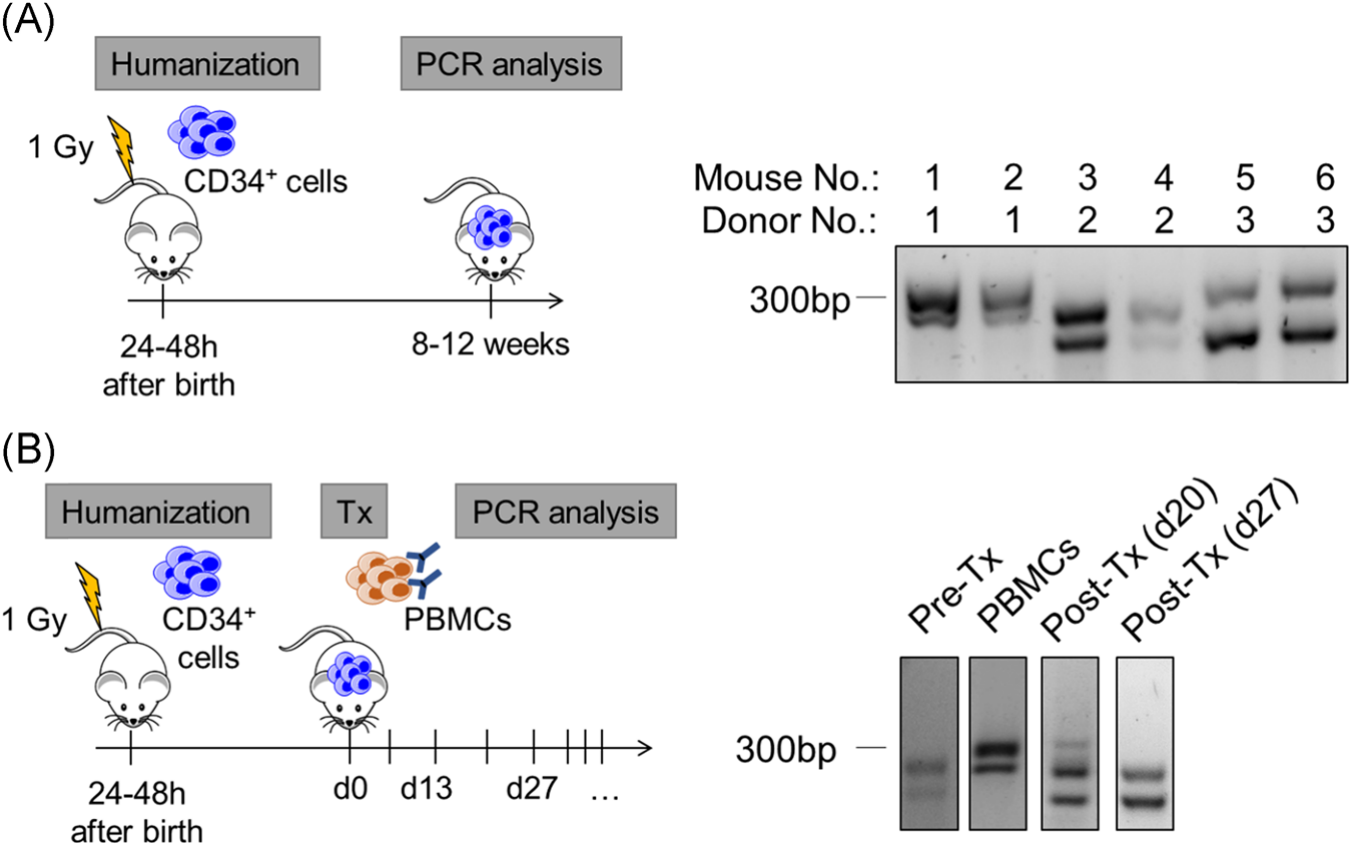
Polymerase chain reaction‐based method for donor‐specific analysis of humanized mice. A, Schematic illustration of humanization (left). Agarose gel electrophoretic analyses of STR motifs (SE33) in peripheral blood DNA samples of humanized mice (right; representative analyses are shown, in total bands were detected in 21 of 21 mice). B, Schematic illustration of humanization followed by transplantation (Tx) of human PBMC (with and without pre‐incubation of MAX.16H5 IgG_4_) (left). The two recipient mice in which the detection of four bands was successful over time (day 20) received a MAX.16H5 preincubated graft. The significance of this observation must be evaluated in further studies. DNA from peripheral blood samples were analyzed by agarose gel electrophoresis for SE33 before and after Tx and compared to DNA from the transplanted PBMC (right). Representative analyses are shown. In total, two donor‐specific bands were detected in 21 of 21 mice, chimerism (ie, four bands) was detected in two of 21 mice. In both panels, n = 21 mice were humanized from n = 13 donors in n = 4 independent experiments. Transplantation of PBMCs was performed in n = 2 independent experiments with n = 10 or n = 11 mice per experiment. bp, base pairs; No., number; PCR, polymerase chain reaction; PBMC, peripheral blood mononuclear cells; STR, short tandem repeats; Tx, transplantation

As many experiments with humanized mice require donor‐specific analyses, we applied a new method: The evaluation of STR fragment lengths by PCR and gel electrophoresis is highly specific, as each human being has a unique combination of STR alleles with different lengths.^21^ Here, we compared STR motifs of mice that were humanized from different CB‐donors and were able to specifically trace back the donors in 21 of 21 mice (Figure 2A). Furthermore, we applied our assay to a humanized transplantation setting, wherein humanized mice were transplanted with human PBMC from a mismatched donor. The STR pattern before transplantation was clearly distinct from the STR pattern of transplanted PBMC. Twenty days after transplantation, four bands were visible, reflecting the two bands from the CB donor as well as the two bands from the PBMC. Shortly after, from day 27 onwards, only the two bands from the CB donor were detected, reflecting a rejection of the PBMC transplant (Figure 2B). Although distinct bands from both donors could be detected in 21 of 21 mice, the intermediate state of chimerism was observed in two of 21 mice.

## 4 CONCLUDING REMARKS

Humanized mice are frequently used in preclinical experiments, especially in the field of immunology.^22^ An efficient method to humanize mice is, therefore, highly relevant. Using adult HSC instead of CB‐derived HSC could open the possibility of analyzing patient‐specific mice in the field of personalized medicine. In our model, despite the in vitro differentiation capacity of adult HSC and the good engraftment in the bone marrow, levels of huCD45 in the PB were below 10% and, therefore, not feasible for a humanized mouse model.

Another objective of this study was to test the feasibility of differentiating between transplanted human cells and cells from the endogenous humanized immune system of the recipient mice. The STR detection method was used to investigate the possibility to distinguish transplanted PBMC (with and without antibody pre‐incubation) with regard to graft rejection or engraftment from the endogenous humanized immune system of the recipients. We successfully demonstrated two applications of PCR‐based STR motif analysis in humanized mice: (a) tracing back, which mice were humanized from which CB donor, and (b) detecting chimerism in experimental settings using two different human donors. Further possible applications could include allogeneic organ and cell transplantation models or allogeneic tumor models. In addition to the high specificity, the assay is fast and requires only basic molecular biological methods and small sample volumes (<30 µL of blood). Other donor identification methods with comparable specificities, such as HLA‐typing, are much more complex and time‐consuming. The STR detection method will be further validated in future transplantation studies using humanized mice.

## CONFLICT OF INTERESTS

The authors declare that there are no conflicts of interests.

## AUTHOR CONTRIBUTIONS

LW, NH, UST, and SF designed the research; LW and NH performed the experiments; LW and ARB analyzed results; LW, NH, AKW, FL, UST, ARB, and SF created figures and wrote the manuscript.

## Data Availability

The data that support the findings of this study are available from the corresponding author upon reasonable request.
